# Ki-67 staining pattern as a prognostic biomarker for advanced acral melanoma^؆^

**DOI:** 10.1016/j.abd.2026.501298

**Published:** 2026-03-19

**Authors:** Marcel Arakaki Asato, Isabeli Joaquim Contel, Francisco Alves Moraes Neto, Juliana Polizel Ocanha-Xavier, Nathália Silva Carlos Oliveira, Maxwell A. Fung, Mariangela Esther Alencar Marques, José Cândido Caldeira Xavier-Júnior

**Affiliations:** a Faculty of Medicine, Universidade Federal do Mato Grosso do Sul, Campo Grande, MS, Brazil; b Faculty of Medicine, Universidade Estadual Paulista, Botucatu, SP, Brazil; c Amaral Carvalho Hospital, Jaú, SP, Brazil; d Faculty of Medicine, Universidade Federal Fluminense, Niterói, RJ, Brazil; e Department of Dermatology, School of Medicine, University of California Davis, Sacramento, CA, United States; f Faculty of Medicine, Centro Universitário Católico Salesiano Auxilium, Araçatuba, SP, Brazil; g Pathology Institute of Araçatuba, Araçatuba, SP, Brazil

**Keywords:** Immunohistochemistry, Ki-67 antigen, Melanoma, Pathology, Skin neoplasms

## Abstract

**Background:**

Acral cutaneous melanoma (ACM) is an aggressive skin cancer, especially when diagnosed in the advanced stage. The Ki-67 is a rapid tool for proliferation rate analysis. Previous data indicated that its staining pattern is distinct during the several stages of the cell cycle among epithelial cells.

**Objective:**

To evaluate the prognostic impact of Ki-67 expression pattern classification among advanced acral cutaneous melanoma cases.

**Methods:**

Two pathologists classified staining nuclear patterns of Ki-67 in the hot spot of scanning slides of advanced ACM (pT4): NP1 (randomly Ki-67 immunopositive fine or coarse granules), NP2 (Ki-67 stained in one or two well defined and centralized nodules), NP3 (Ki-67 seen as granules or nodules occupying most of the nucleus), NP4 (nucleoplasm with intense and homogeneous Ki-67 staining) and NP5 (empty central area with peripheral Ki-67). For analysis, seven cases per group ‒ Alive (Al) and Melanoma-Related Death (De) ‒ were randomly selected.

**Results:**

This pilot study analyzed 5676 Ki-67 positive nuclei. Means comparison between the two groups revealed differences in nuclear patterns NP1 (p = 0.0014), NP4 (p = 0.038), NP5 (p = 0.0193), and the total number of stained nuclei (p = 0.0258).

**Study limitations:**

Small number of cases and biased value of 6.35 through the Bland-Altman analysis.

**Conclusion:**

The present analysis highlights the importance of Ki-67 staining patterns as a potential prognostic marker among ACM. High Ki-67 positive nuclei were associated with worse outcomes (death), particularly in staining patterns before and after mitosis (NP1, NP4, and NP5).

## Introduction

Cutaneous melanoma is an aggressive skin cancer^1^, ^2^ with Breslow depth recognized as the most important criterion for staging.^3^, ^4^ The latest World Health Organization classification of skin tumors reflects the most up-to-date understanding of melanoma subtypes, incorporating not only microscopic characteristics but also molecular features and potential pathways of carcinogenesis.^5^ In this classification, acral cutaneous melanomas (ACM) are considered as a distinct group.^5^ It was first described by Reed in 1976 (apud Kuchelmeister et al.),^6^ with a higher prevalence in African and Asian populations and typically found on palms, soles, and nail beds.^7^, ^8^ Notably, ACM is associated with poorer outcomes when compared with the other melanoma subtypes, even after adjusting for tumor thickness.^7^

Immunohistochemical markers complement the histopathological analysis for the diagnosis and outcomes of many types of cancer. However, no markers for cutaneous melanoma have been required for diagnosis or staging protocols.^9^, ^10^ Evidence from multiple studies indicated Ki-67 as a prognostic marker in cutaneous melanoma, as elevated Ki-67 indices correlate with unfavorable outcomes.^11^, ^12^, ^13^, ^14^, ^15^

The Ki-67 is a cell proliferation marker, with nuclear expression in the cell cycle's G1, S, G2, and mitotic phases, but it is absent in G0 cells. Therefore, it is a simple and rapid tool for assessing tumor proliferation.^16^ Previous studies have demonstrated that Ki-67 has distinct nuclear localization patterns during the several stages of the cell proliferation cycle.^17^, ^18^ It localizes to the perichromosomal layer at the beginning of mitosis, to hundreds of distinct foci homogeneously distributed in the early G1 phase, to one or two nucleoli in the late G1 phase, to larger dense granules throughout the nucleus in the S phase, and granules and irregular nodules in the nucleoplasm in the G2 phase.^19^, ^20^ Dias et al., in 2021, created a classification of cell cycle phases based on the morphological Nuclear Patterns (NP) of Ki-67 immunostaining. Based on the analysis of epithelial tumors, they also found differences in the pattern of Ki-67 expression between benign epithelial lesions and malignant or pre-malignant epithelial lesions.^20^

To date, no studies have evaluated the pattern of Ki-67 expression in cutaneous melanoma. This study aims to analyze the interobserver concordance of Ki-67 expression pattern classification in ACM and to compare the different types of patterns between two groups of patients: those who are alive (Al) and those who died of melanoma (De).

## Methods

This is a retrospective and cross-sectional pilot study that analyzed formalin-fixed paraffin-embedded skin specimens collected from advanced ACM, all classified as the same stage (pT4),^10^ from a Brazilian referral cancer center. This study defined the inclusion criteria as cases of invasive pT4 ACM diagnosed between 2008 and 2020, given that this stage group is associated with the poorest outcomes. Exclusion criteria included cases with failed or suboptimal immunohistochemical staining, absence of survival data, and ungual melanomas. For analysis, seven cases per group ‒ Alive (Al) and Melanoma-Related Death (De) ‒ were randomly selected. Nuclei that were overlapping or blurred were not included in the evaluation. Patients’ follow-up duration was described by group in months.

**Fig. 1:**
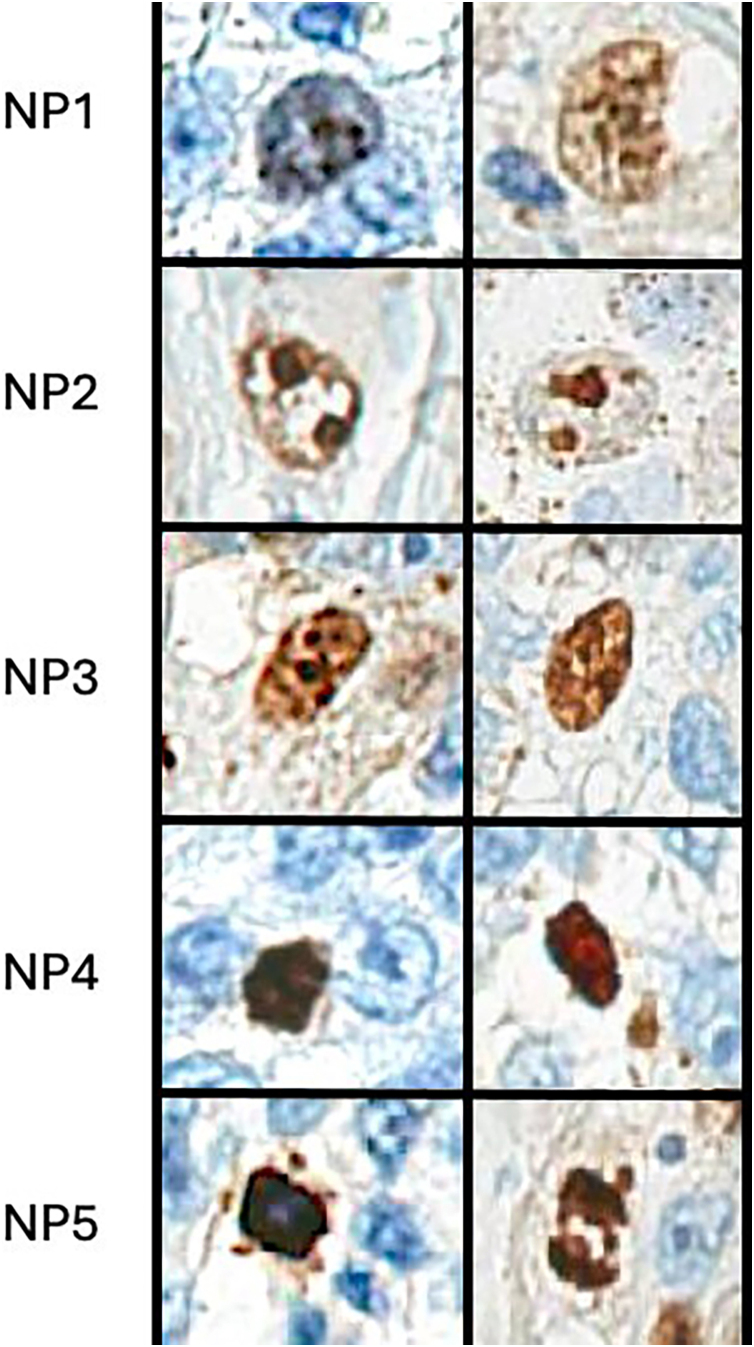
Examples of each Ki-67 staining pattern, based on Dias’ classification.

**Fig. 2:**
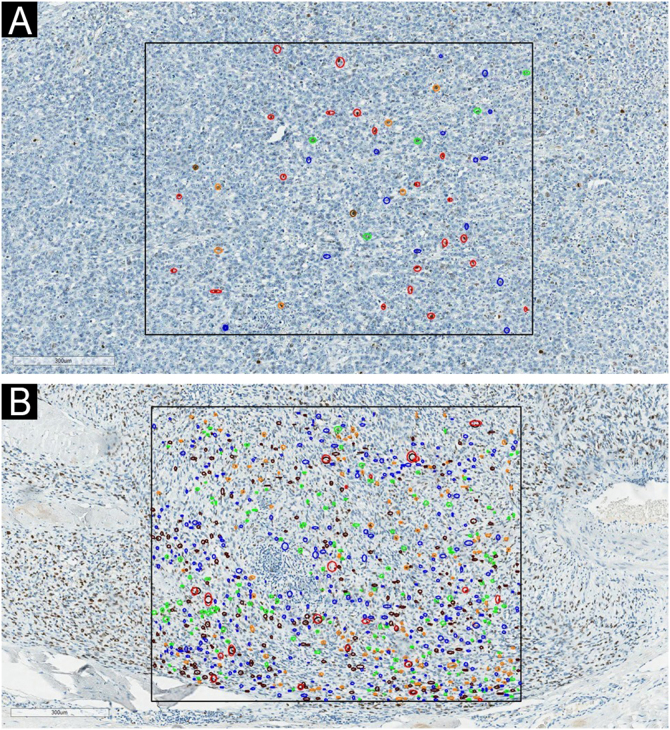
Examples of the one-square-millimeter demarcated area and the classification of Ki-67 staining patterns through colored circles. A case of “Al” group in image A and “De” group in image B. Blue circle: NP1. Green circle: NP2. Orange circle: NP3. Brown circle: NP4. Red circle: NP5.

Ventana Roche performed Immunohistochemical (IHC) analysis on the Benchmark GX automatic staining system, applying the UltraView DAB kit using Ki-67 (Dako, clone MIB-1, prediluted). The immunohistochemistry slides, sectioned at 3 µm, were scanned using the Aperio CS™ system (Leica Biosystems, USA). A one-square-millimeter section was demarcated in hot spots for a detailed examination of each sample. This area size was chosen to be aligned with AJCC mitosis counting.^10^ The slides were evaluated by two pathologists, independently identifying Ki-67 positive nuclei within the designated area (hot spot), utilizing a colored circle scheme based on Dias’ classification, as follows: blue circle for NP1 (randomly Ki-67 immunopositive fine or coarse granules, corresponding to early G1 phase), green circle for NP2 (Ki-67 stained in one or two well defined and centralized nodules, corresponding to late G1 phase), orange circle for NP3 (Ki-67 seen as granules or nodules occupying most of the nucleus, corresponding to S phase), brown circle for NP4 (nucleoplasm with intense and homogeneous Ki-67 staining, corresponding to G2 phase) and red circle for NP5 (empty central area with peripheral Ki-67 or exclusively on the chromosomes, corresponding to mitosis) as shown in Fig. 1, Fig. 2. Then, the number of stained nuclei was counted for each pattern in all cases. Only nuclear positivity was considered; cytoplasmic or membranous staining was not included. Both pathologists analyzed exactly the same area, but the nuclei to be counted were not previously marked. They were previously trained using five epithelial tissue samples obtained from the research group’s archives. To assess the agreement between measurements, a Bland-Altman graphical analysis was conducted to calculate the bias between measurements, Lin's Concordance Correlation Coefficient, and the corresponding plot. Outcome measures were correlated with survival data extracted from electronic health records, leading to the categorization of the cases into two groups: those who were alive and those who died of melanoma.

This analysis was performed using the STATA software for Windows, version 21. An average of each evaluator's data was computed. A comparison of means for each nuclear staining pattern was conducted, utilizing a negative binomial distribution adjustment.

A significance threshold of 5% or the corresponding p-value was established for all tests. The analyses were conducted using SAS for Windows version 9.4. and SPSS for Windows version 21. To evaluate the risk of bias, the Study Quality Assessment Tool for Cross-Sectional Diagnostic Pathology Studies (SQAT-Path),^21^ adapted from the modified Newcastle–Ottawa scale, was applied.

This research received approval from the research ethics committee (CAAE 52618721.0.1001.5411 and CAAE 52618721.0.3001.5434).

## Results

**Fig. 3:**
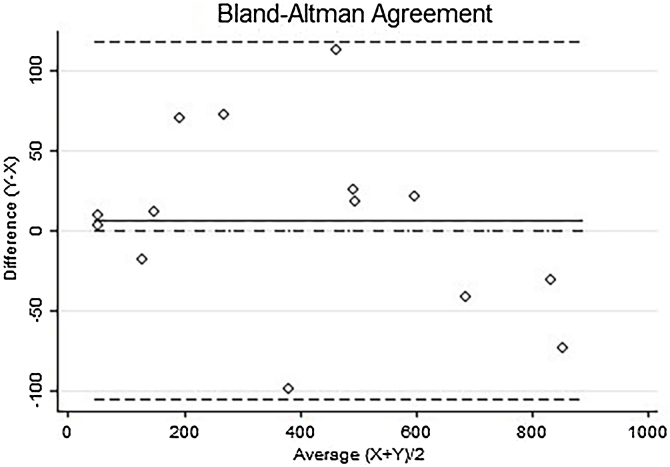
Bland-Altman agreement plot of the analysis of the nuclear patterns between two groups (alive patients and patients with acral melanoma-related death, n = 14) performed by two pathologists. On the abscissa (X) axis are the means of positive nuclei of each case performed by the two evaluators, and on the ordinate (Y) axis is the difference between positive nuclei counts.

In this analysis, a total of 5676 Ki-67 positive nuclei from 14 cases were evaluated, with 7 cases in the “AI” group and 7 cases in the “De” group. In both the “AI” and “De” groups, 4 cases (57.15%) were male, while 3 cases (42.86%) were female. The mean age of the “AI” group was 65-years, whereas in the “De” group, the average age was 74-years. Regarding Breslow thickness, the mean Breslow thickness in the “AI” group was 7.2 mm (SD = 1.6; median: 7.4 mm; range: 4.3–9.3), while in the “De” group, the mean Breslow thickness was 9.1 mm (SD = 2.08; median: 8.1 mm; range 7–13). The “AI” group had a mean follow-up duration of 52-months, while the “De” group demonstrated a mean survival of 31-months following diagnosis. Considering the application of the digital cell counting based on Ki-67 staining pattern in the hot spot, patients from group “Al” exhibited a total of 1689 Ki-67-positive nuclei, in contrast to the “De” group, who presented with a total of 3987 positive nuclei (Table 1). Additionally, a significant difference was noted in the total number of positive nuclei (p = 0.0258), with the “Al” group having a mean of 241.21 (SD = 184.47, range: 51–493.5) and the “De” group having a mean of 569.57 (SD = 258.45, range: 130–855) (Table 2). Lin's Concordance Correlation Coefficient of Absolute Agreement was reported at 0.9783, reflecting a substantial level of agreement among pathologists ^22^. The Bland-Altman analysis revealed a bias of 6.36 with a 95% Confidence Interval from -26.61 to 39.32 (SD = 57.10) as shown in Fig. 3.

**Table 1:** Ki-67 index and quantification of Ki-67 staining pattern across the 14 cases.

Alive group	Ki-67 index	Ulceration	NP1	NP2	NP3	NP4	NP5	Total
Case 1	20%	Absent	56 (29.3%)	46 (24.0%)	81 (42.4%)	5 (2.6%)	3 (1.6%)	191
Case 2	5%	Present	18 (35.2%)	5 (9.8%)	11 (21.5%)	2 (3.9%)	15 (29.4%)	51
Case 3	5%	Absent	20 (37.7%)	9 (16.9%)	19 (35.8%)	2 (3.7%)	3 (5.6%)	53
Case 4	60%	Present	65 (23.9%)	81 (29.8%)	112 (41.3%)	10 (3.6%)	3 (1.1%)	271
Case 5	50%	Absent	24 (5.0%)	331 (68.9%)	121 (25.2%)	3 (0.6%)	1 (0.2%)	480
Case 6	10%	Present	72 (48.3%)	23 (15.4%)	48 (32.2%)	5 (3.3%)	1 (0.6%)	149
Case 7	40%	Present	98 (19.8%)	38 (7.6%)	271 (54.8%)	86 (17.4%)	1 (0.2%)	494
Total (%)	27.1%		353 (20.8%)	533 (31.5%)	663 (39.2%)	113 (6.6%)	27 (1.59%)	1689
De^a^ group	Ki-67 index	Ulceration	NP1 (%)	NP2 (%)	NP3 (%)	NP4 (%)	NP5 (%)	Total
Case 1	10%	Present	191 (38.5%)	55 (11.0%)	208 (41.9%)	38 (7.6%)	4 (0.8%)	496
Case 2	5%	Present	136 (35.5%)	76 (19.8%)	101 (26.3%)	25 (6.5%)	45 (11.7%)	383
Case 3	40%	Present	226 (27.0%)	141 (16.8%)	392 (46.8%)	61 (7.2%)	16 (1.9%)	836
Case 4	30%	Present	132 (22.0%)	175 (29.2%)	258 (43.0%)	26 (4.3%)	8 (1.3%)	599
Case 5	5%	Absent	31 (23.8%)	18 (13.8%)	50 (38.4%)	30 (23.0%)	1 (0.7%)	130
Case 6	60%	Absent	241 (28.1%)	120 (14.0%)	316 (36.9%)	170 (19.8%)	8 (0.9%)	855
Case 7	50%	Absent	191 (27.7%)	216 (31.3%)	216 (31.3%)	43 (6.2%)	22 (3.1%)	688
Total (%)	28.6%		1148 (28.7%)	801 (20%)	1541 (38.65%)	393 (9.8%)	104 (2.6%)	3987

a De, Melanoma-related death.

**Table 2:** Means comparison of Ki-67 staining patterns between the two groups.

Alive group					Deceased group				
	Total (%)	Mean	SD	Median (min‒max)	Total (%)	Mean	SD	Median (min‒max)	p-value
NP1	353 (20.9%)	50.43	30.68	56 (18–98)	1148 (28.8%)	164	71.56	191 (31–241)	**0.0014**
NP2	533 (31.6%)	76.14	115.28	38 (5–331)	801 (20.1%)	114.43	69.56	120 (18–216)	0.4322
NP3	663 (39.3%)	94.71	88.67	81 (11–271)	1541 (38.7%)	220.14	117.94	216 (50–392)	0.051
NP4	112.5 (6.6%)	16.07	30.74	5 (2–85.5)	393 (9.8%)	56.14	51.72	38 (25–170)	**0.038**
NP5	27 (1.6%)	3.86	5.01	3 (1–15)	104 (2.6%)	14.86	15.08	8 (1–45)	**0.0193**
Total	1688.5 (100.0%)	241.21	184.47	191 (51‒493.5)	3987 (100.0%)	569.57	258.45	599 (130–855)	**0.0258**

A comparative analysis of means between the two groups revealed statistically significant differences in nuclear staining patterns NP1 (p = 0.0014), NP4 (p = 0.038), and NP5 (p = 0.0193). Specifically, the “Al” group had a mean of 50 nuclei classified as NP1 (SD = 30.68, range: 18–98). The “De” had a mean NP1 of 164 (SD = 71.56 range: 31–241). For the NP4 pattern, the “Al” group's mean was 16 nuclei (SD = 30.74 range: 2–85.5), whereas the “De” group had an NP4 mean of 56.14 (SD = 51.72 range: 25–170) As for NP5, the mean for the “Al” group was 3.86 (SD = 5.01 range: 1–15), compared to the “De” group, which had a mean NP5 of 14.86 (SD = 15.08 range: 1–45). No significant differences were found for NP2 and NP3 patterns (p = 0.4322 and p = 0.051, respectively).

## Discussion

Our analysis highlights the possible relevance of Ki-67 staining patterns as a prognostic indicator among advanced ACM. Lin's Concordance Correlation Coefficient was high, in agreement with Dias et al., who showed a moderate diagnostic interobserver agreement in their study (κ = 0.523). Significant differences in nuclear staining patterns were observed between the two analyzed groups. Higher counts of Ki-67 positive nuclei were associated with worse outcomes (death), particularly in staining patterns NP1 (early G1 phase), NP4 (G2 phase), and NP5 (mitosis), which may indicate more aggressive tumor behavior. Since these three patterns are closely related to the mitotic process (NP4 before, NP5 during, and NP1 after mitosis), which is a well-established biomarker of aggressiveness, the results reinforce the applicability of the proposed methodology and highlight its relevance in melanoma cases

Previous studies showed that a high proliferation is associated with a worse prognosis.^11^, ^12^, ^13^ Indeed, until 2015, mitosis was a criterion for staging in the seventh edition of AJCC Melanoma Staging.^23^ Ki-67 is a well-established prognostic parameter in melanoma patients,^24^ and based on these data, the authors suggest that Ki-67 analysis may have potential applicability beyond simply estimating the number of positive cells.

Regardless of prognosis, Dias et al. showed that among malignant or pre-malignant epithelial lesions NP4 nuclei predominated, while among benign lesions, NP1 and NP2 nuclei predominated.^20^ Our study, however, did not encounter a pattern that was predominant in “Al” (alive) patients with ACM. NP1, NP4, and NP5 patterns were more frequent in the “De” (melanoma-related death) group. Loddo et al. studied cell-cycle-phase progression in 182 patients with invasive breast cancer. They found that increased tumor grade is associated with an increased proportion of cells engaged in the cell division cycle (G1 – S and G2 – M regulators).^23^

The majority of neoplasms exhibit a dysregulated cell cycle due to alterations in the regulators of CDK function. Additionally, many oncogenes contribute to this dysregulation of the cell cycle.^25^ The identification of the cell cycle phase could play an important role in therapeutic strategies, given that a variety of anti-cancer agents, including those used for melanoma, not only affect the cell cycle but are also dependent on specific phases to be effective. Bortezomib, for example, induces G1 and G2 arrest and causes apoptosis of G2 phase cells.^26^, ^27^ CDK4/6 inhibitors block progression out of the G1 phase. Gemcitabine acts during the S phase.^28^ Flavopiridol acts in the G1 and G2 phases, causing cell cycle arrest and preventing cells from entering the M-phase. Paclitaxel is more active in the M-phase.^29^

Furthermore, certain drugs target highly proliferative cells more effectively than those in slow cycling.^26^ The present study showed that most classified cells were situated before mitosis, and the time required for cells to reach mitosis after initial Ki-67 expression can vary based on the cell cycle speed. This finding may explain the possible failure of more active drugs in the M-phase. It can also corroborate the idea of mitotic count as a suboptimal parameter to predict outcomes. Considering the importance of the cell cycle and the action of drugs on it, classifying the nuclear patterns of Ki-67 to determine the phase of tumor cells could provide a personalized patient report plan to guide oncologists’ conduct and unlock new possibilities for treatment optimization. However, further studies are needed to standardize procedures, establish clinical protocols, and evaluate the applicability of artificial intelligence in this context.

Previous studies established Ki-67 as a prognostic marker in cutaneous melanoma, but until now, no studies have investigated and compared the specific staining patterns. The present study has some limitations. A limited number of cases was evaluated (although a total number of 5676 nuclei is remarkable), and the study was restricted to the ACM subtype in the advanced stage identified from a single institution. The bias value of 6.35 regarding observers’ analysis was notable, although Lin's Concordance Correlation Coefficient indicated a high level of agreement. The high bias may be attributable to interpersonal subjectivity in pattern assessment. Furthermore, the absence of pre-selection of nuclei for analysis could contribute to the variability, although it made the analyses closer to the routine practice scenario (real world). Nevertheless, when evaluated with the SQAT-Path^21^ the study demonstrated a low risk of bias.

These findings highlighted the applicability of Ki-67 nuclear staining patterns to predict outcomes among advanced ACM cases. Further studies with more significant cases are needed to elucidate the differences in Ki-67 staining patterns across various cutaneous cancer types, their correlation with patient outcomes and chemotherapy response, and to compare these findings with existing measures of melanoma prognosis such as mitotic index. These results open new perspectives for developing computer algorithms to assist pathologists in analyzing ACM prognosis more comprehensively than by simply estimating the percentage of Ki-67–positive cells.

## Authors' contributions

Marcel Arakaki Asato: Investigation; data curation; formal analysis; writing-original draft.

Isabeli Joaquim Contel: Investigation; data curation; formal analysis; writing-review & editing.

Nathália Silva Carlos Oliveira: Conceptualization; review & editing.

Francisco Alves Moares Neto: Formal analyses and review & editing.

Mariangela Esther Alencar Marques: Review & editing.

Juliana Polizel Ocanha-Xavier: Conceptualization; review & editing.

Maxwell A. Fung: Writing-review & editing.

José Cândido Caldeira Xavier-Júnior: Conceptualization; methodology; investigation; writing-review & editing; supervision.

## Ethics statement

The committees of ethics in research at the Faculdade de Medicina – UNESP/Botucatu – SP (CAAE 52618721.0.1001.5411) and the Amaral Carvalho Hospital (CAAE 52618721.0.3001.5434) approved the study. Patient consent not applicable.

## Abbreviations and ORCID:

Marcel Arakaki Asato: 0000-0002-6050-5292

Isabeli Joaquim Contel: 0000-0001-6943-338X

Francisco Alves Moares Neto: 0009-0001-7820-8106

Juliana Polizel Ocanha-Xavier: 0000-0002-1200-3730

Nathália Silva Carlos Oliveira: 0000-0003-3733-0022

Maxwell A. Fung: 0000-0003-1771-8257

Mariangela Esther Alencar Marques: 0000-0001-6947-5627

## Financial support

Fundação de Amparo à Pesquisa do Estado de São Paulo, Brazil. The funder had no role in the design, data collection, data analysis and reporting of this study.

## Research data availability

The entire dataset supporting the results of this study was published in this article.

## Conflicts of interest

None declared.
